# Effects of Exercise on EEG Activity and Standard Tools Used to Assess Concussion

**DOI:** 10.1155/2019/4794637

**Published:** 2019-04-30

**Authors:** David M. Devilbiss, Jena L. Etnoyer-Slaski, Emily Dunn, Christopher R. Dussourd, Mayuresh V. Kothare, Stephen J. Martino, Adam J. Simon

**Affiliations:** ^1^Rowan University, Department of Cell Biology and Neuroscience, 2 Medical Center Drive, SC220, Stratford, NJ 08084, USA; ^2^Lehigh University, Department of Sports Medicine, 641 Taylor Street, Bethlehem, PA 18015, USA; ^3^Lehigh University, Department of Chemical & Biomolecular Engineering, B323/D322 Iacocca Hall, 111 Research Drive, Bethlehem, PA 18015, USA; ^4^Monmouth-Ocean Neurology, 1944 State Route 33, Suite 206, Neptune, NJ 07753, USA; ^5^Cerora Inc., 116 Research Drive, Bethlehem, PA 18015, USA

## Abstract

A variety of cognitive assessment tools are used to determine the functional status of the brain before and after injury in athletes. Questionnaires, neuropsychological tests, and electroencephalographic (EEG) measures have been recently used to directly assess brain function on and near the playing field. However, exercise can affect cognitive performance and EEG measures of cortical activity. To date, little empirical evidence exists on the effects of acute exercise on these measures of neurological function. We therefore quantified athlete performance on a standardized battery of concussion assessment tools and EEG measurements immediately before and after acute exercise to simulate conditions of athletic competition. Heart rate and arterial oxygen levels were collected before and after the exercise challenge consisting of a 1-mile run. Together these data, from a gender-balanced cohort of collegiate athletes, demonstrated that moderate to hard levels of acute exercise improved performance on the King-Devick test (K-D test) and Standardized Assessment of Concussion (SAC) component of the Sport Concussion Assessment Tool (SCAT3). Gender played an important role in these effects, and performance was most affected by exercise in female athletes. EEG activity in the theta band (4–8 Hz) was decreased during periods of quiet resting with eyes open or eyes closed. Additionally, exercise produced a slowing of the EEG during the K-D test and a shift to higher frequencies during the balance assessment of the SCAT3. Together, these data indicate that exercise alone can influence outcome measures of cognitive assessment tools used to assess brain function in athletes. Finally, care must be taken to acquire postinjury measurements during a comparable physiologic state to that in which baseline assessment data were measured, and further research is needed into the factors influencing outcome measures of these tests.

## 1. Introduction

Exercise, including short bouts of running, affects a wide range of cognitive processes [1]. Information processing speed, reaction time, and “executive” cognitive function, including working memory, attention, planning, and behavioral inhibition, represent several processes that are improved with acute exercise [2–6]. Beyond changes in cognitive function, exercise also increases cortical activity levels as measured by electroencephalography (EEG) [7]. EEG recordings measure the average electrical activity from large populations of neurons in the cortex over time and reflect the ongoing neural computations and information processing of the brain [8]. The EEG signal contains a spectrum of frequencies that have been phenomenologically divided into several increasing frequency ranges or bands including delta (1–4 Hz), theta (4–8 Hz), alpha (8–12 Hz), beta (12–30 Hz), and gamma (>30 Hz). Different combinations of these rhythms are associated with either improvements or impairments in cognitive function. Moreover, limited evidence indicates that the EEG spectrum may differ before and after acute exercise, including changes in the power of the alpha and beta range of the EEG spectrum [7, 9, 10]. However, the relationship between improved cognitive function following exercise and changes in EEG activity are poorly understood.

Concussion is a type of mild traumatic brain injury (mTBI) that affects approximately 1.7 million Americans each year [11, 12]. Actual incidence rates may be as high as 3.8 million when including cases not seen by emergency department physicians [13]. The symptoms of concussion and mTBI may include a rapid onset of a number of graded clinical symptoms characterized by impairments in information processing, delayed visual motor reaction time, and reduced performance on attention and memory tasks [14–17]. A variety of cognitive assessment tools are used to assess these cognitive processes including the Sport Concussion Assessment Tool (SCAT3) [12, 18] and/or the King-Devick neuro-ophthalmologic saccade test (K-D test) [19]. These assessments are typically performed before the athletic season (baseline) and after a suspected injury (postconcussion). Baseline testing is frequently administered in a quiet and controlled setting, whereas postconcussion testing is performed immediately after play/exercise, frequently “on field” or “on-site” [12]. Additionally, quantitative EEG (qEEG) is beginning to be used as a direct measure of changes in cortical function following concussion [20]. We have shown that portable, single channel EEG devices can be used “on field” or “on-site” as an objective measure of cortical activity changes following concussion [21–23]. Evidence from both cognitive testing and EEG measures suggests that these data may be affected by exercise [1]. Yet, the impact of exercise on these assessment tool measures of cognitive function is poorly understood.

In the current set of experiments, we determined the degree to which exercise alters baseline measures of cognitive function in commonly used concussion assessment tools and qEEG measures of cortical activity using portable EEG devices. We found that, in a gender-balanced cohort of collegiate athletes, moderate to hard levels of acute exercise improved performance in the SCAT3 and K-D test. Performance in female athletes was affected more than males by acute exercise. EEG activity in the theta band was reduced following exercise in periods of quiet resting with eyes open or eyes closed. Finally, exercise produced a slowing of the EEG during the K-D test and a shift to higher frequencies during the balance assessment of the SCAT3. Together, these data suggest that exercise alone can influence concussion assessment tool measures of brain function. Furthermore, these data indicate that care must be taken to acquire postinjury measurements during a comparable physiologic state to that in which baseline assessment data were measured.

## 2. Methods and Materials

### 2.1. Participants

Healthy athletes from Lehigh University men's and women's collegiate soccer team volunteered for this study (*n* = 16; Supplemental Table 1, subject demographic profile). Participants included both goalies and field players. Similar demographic backgrounds (age, race, and handedness) were self-reported across all subjects. Importantly, subjects were not permitted to have experienced a concussion or mTBI within the prior two months for inclusion in this study. Additional exclusion criteria included any history of stroke, seizures, or chronic pain, a diagnosis of moderate to severe TBI at any point in the subject's life, or current use of sedatives or narcotics.

The current study included a single device, single visit, parallel group experimental design that was approved by the Lehigh University Institutional Review Board (IRB). All subjects were required to complete informed consent and IRB forms prior to participation in the study. Subjects were assigned a random/unique ID, and all electronically collected study data were encrypted (AES-256) before transmission to analysis computers. Personal health information and other identifying information were retained at Lehigh University Sports Medicine in a secure, locked storage facility.

### 2.2. Procedures

**Figure 1:**
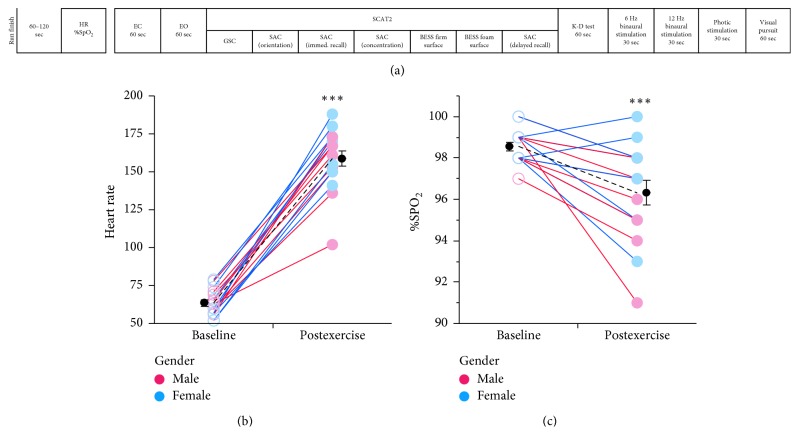
Time course of the 14-task neural assessment protocol and the effect of exercise on heart rate and arterial oxygen levels in collegiate athletes. (a) The 14-task neural assessment protocol conducted before and after the exercise challenge. (b) Heart rate (beats/minute) is plotted before (baseline) and after (postexercise) a timed one-mile run around a grass track. Males (*n* = 8, red) and females (*n* = 8, blue) showed similar 250% elevations in heart rate. (c) Following exercise, the average arterial oxygen level (% SpO_2_) was decreased 2% for both males and females. Data for each subject are plotted individually; group means ± SEM are plotted in black. ^*∗*^ *p* < 0.05; ^*∗∗*^ *p* < 0.01; ^*∗∗∗*^ *p* < 0.001 Wilcoxon test.

All subjects underwent an identical assessment protocol immediately before and after a demanding 1-mile run. During the assessment protocol, subjects were seated comfortably in a chair facing a laptop PC. Resting heart rate (HR) and percent oxygen saturation (%SpO_2_) as measured by using a finger pulse oximeter (Nonin, Model Onyx II 9560) were recorded while the EEG recording headset (Cerora Borealis, Bethlehem, PA; investigational device not yet approved by the FDA) was positioned on the subject's head. EEG was recorded during the completion of a 14-task neural assessment protocol. Immediately after the baseline assessment, subjects were requested to remove the EEG headset and run a mile speed test. Subjects were requested to run the mile at their individual best time (i.e. race pace) on a grass track surface. HR and %SpO_2_ were measured within 1-2 minutes of completion of the mile speed test. The 14-task neural assessment protocol and EEG recording were then administered immediately following HR and %SpO_2_ measures (within approx. 30 seconds; Figure 1(a)).

The 14-task neural assessment protocol included (i) an eyes-closed (EC) resting condition for 60 seconds and (ii) an eyes open (EO) resting condition for 60 seconds. These were followed by a subset of components from the SCAT3 instrument including (iii) the 22-item graded symptom checklist (GSC), (iv) Standard Assessment of Concussion- (SAC-) orientation task, (v) SAC-immediate memory recall task (5-word immediate recall in three trials), (vi) SAC-concentration task (repeating single numbers back in reverse order), and the (vii) full balance error scoring system (BESS) task. During the BESS component of the assessment protocol, subjects were required to stand and engage in three static postures (double leg stance, single leg stance, and tandem stance [24]). The BESS task was re-administered (viii) on a foam surface (2′ × 2′ × 4″ thick Airex foam pad), repeating the three stances. This task was immediately followed by the (ix) SAC-Delayed Recall task, the (x) the King-Devick neuro-ophthalmologic saccade test (K-D test [19]), a continuous dichotic auditory stimulation task (50–75 dB, 30 seconds) with binaural beat frequency of (xi) 6 Hz (400 ± 3 Hz) and (xii) 12 Hz (400 ± 6 Hz), (xiii) a photic stimulation task (5 Hz black-white sinusoid of increasing/decreasing luminosity, 30 seconds), and (xiv) an ocular-visual pursuit task (i.e., visually tracking a crosshair displayed on a monitor with a fixed head position, 60 seconds). On average, the assessment protocol was completed within 15 minutes.

### 2.3. EEG Data Acquisition and Signal Processing

#### 2.3.1. Acquisition

Single-channel EEG was recorded near position Fp1 in accordance with the international 10/20 system [25]. The monopolar electrode was positioned with a physical armature extending from the left ear with the electrical reference and ground located on the left ear. Passive dry stainless-steel electrodes were used for recording, reference, and ground electrodes. Signals were high-pass filtered (cutoff 1.0 Hz) and notch filtered (60 Hz) prior to sampling at 125 Hz with a 10 bit ADC within the headset circuitry (TGEMv2 headset NeuroSky, San Jose, CA). Continuous EEG activity was recorded as individual epochs during each of the 14 tasks (Cerora Borealis ver. 1.5, Cerora Inc., Bethlehem, PA). Subject response data from the neuropsychological and psychological testing battery were simultaneously collected by both the Cerora Borealis software and manually on paper by the test administrator. Finally, EEG and subject response data (mouse, keyboard, and microphone) were encrypted locally (SilverKey; Inv Softworks LLC Seattle, WA) before being transferred and stored in a private cloud for long-term HIPAA compliant storage.

#### 2.3.2. Preprocessing and Denoising

Line noise was effectively eliminated from EEG recordings through the use of a wireless, battery-powered EEG recording device and notch filtering. EEG signal DC offsets were removed independently for each recording epoch. Large amplitude artifacts (greater than 4.5 standard deviation) were identified and replaced with surrogate data derived from FFT interpolation of preceding and trailing data [22, 26]. This approach successfully removed the majority of eye-blink artifact and other large amplitude artifacts while preserving the power spectrum (Supplemental Figure 1). Finally, recordings during each epoch were trimmed to be equivalent in length. Data collected beyond the length of the shortest recording segment were discarded.

#### 2.3.3. Signal Processing and Feature Extraction

EEG time series data were transformed into the frequency domain (0.25 Hz resolution) by applying a fast Fourier transformation (FFT; 8 second data segment with 0.5 second overlap) and Blackmann windowing. In addition to absolute power measures, relative power was calculated for each time point and frequency as a function of the cumulative power across all frequencies for a given point in time. Average absolute or relative power for each traditional EEG band (delta, 1–4 Hz; theta, 4–8 Hz; alpha, 8–12 Hz; beta, 12–30 Hz; gamma, >30 Hz) was found by taking the geometric mean across each 0.25 Hz frequency bin in a band range. Direct ratios of band power were used as an extended set of qEEG band ratio features. Within each task, power and band ratio values were represented as the time-averaged geometric mean of each FFT data segment across time.

### 2.4. Statistics

In the current study, the univariate effects of exercise (pre vs post) on physiologic parameters, cognitive test scores, and qEEG features were initially tested with a Wilcoxon signed-rank test (nonparametric paired *t*-test). Within each set of measures (physiologic, cognitive, and qEEG), false discovery rate (FDR) adjustment was used to correct for multiple comparisons and control type I error [27].

Multivariate interactions of exercise and physiologic measures (gender, age, heart rate, and %SpO_2_) on qEEG relative power measures and band ratios were tested with ANOVA or ANCOVA models. Relative power in band ratios as well as band ratio power was found to be normally distributed in the majority of variables when separated by subject gender and task component. Any violations of normalcy in these data were minimal after 10 data points were removed as outliers. The Tukey–Kramer HSD test was used post hoc with specific effects tests (*t*-tests) to identify specific statistical differences. All analyses were conducted in JMP Pro v11 software (SAS, Inc., Cary, NC).

## 3. Results

Completion of a timed 1-mile run lasted 5–10 minutes and produced significant increases in heart rate and decreases in %SpO_2_ (Figure 1). Heart rate across athletes was increased 2.5-fold (*n* = 16, 63.7 ± 2.1 to 158.7 ± 5.2 bpm (mean ± SEM); Wilcoxon *p* < 0.001) resulting in rates that were 82% of HR_max_ (mean HR/(208– (0.7 ∗ mean age))) [28]. Similar increases were observed for both males (*n* = 8, 65.3 ± 2.5 to 151.1 ± 8.2 bpm; Wilcoxon *p*=0.004) and females (*n* = 8, 62.1 ± 3.5 to 166.3 ± 5.6 bpm; Wilcoxon *p* < 0.039) (Figure 1(b)). Following acute exercise, arterial oxygen levels (%SpO_2_) were decreased 2% across all subjects (*n* = 16, 0.98-fold; 98.6 ± 0.20 to 96.6 ± 0.59; Wilcoxon *p* < 0.001). The decrease in %SpO_2_ after exercise was also similar between males (98.5 ± 0.3 to 95.8 ± 0.8; Wilcoxon *p*=0.004) and females (98.6 ± 0.26 to 96.9 ± 0.8; Wilcoxon *p*=0.047) (Figure 1(c)).

### 3.1. Exercise Effects on Cognitive and Vestibular Tasks

**Figure 2:**
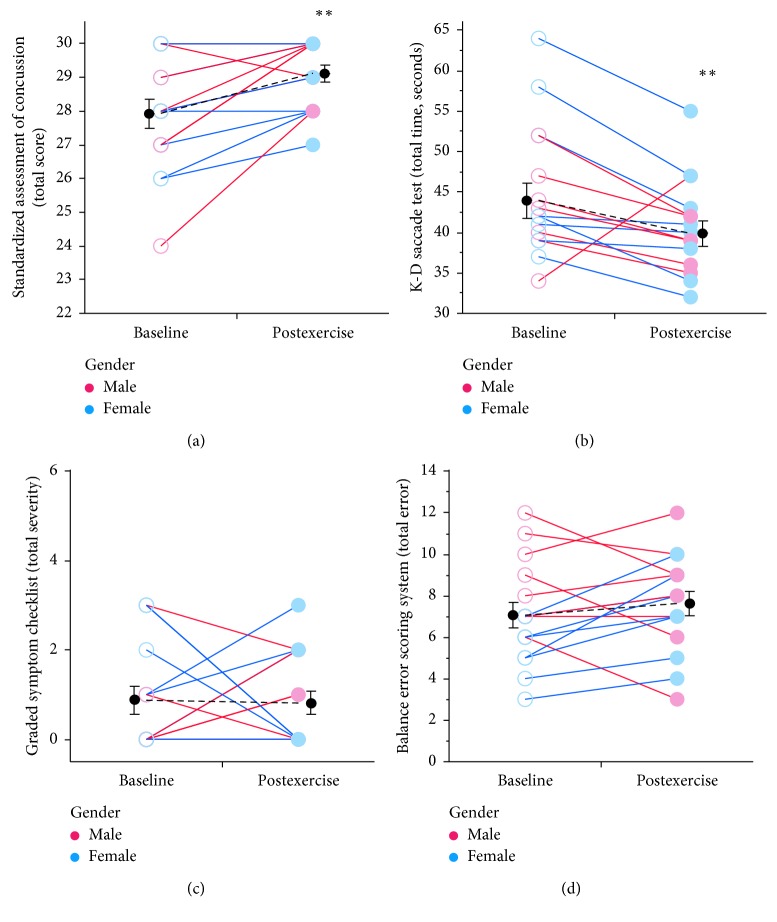
Effect of moderate exercise on four concussion inventories. (a) Performance on the SAC before (baseline) and after (postexercise) exertion. (b) Performance on the K-D test following exercise was also improved (decreased) for all subjects. Female subjects demonstrated the most change from baseline (see main text). (c) Total severity score of the GSC questionnaire (max = 132) was not changed after exercise. (d) Exercise did not significantly alter performance on the BESS (total errors across six postures) for all subjects. Data were collected from the same subject groups (*n* = 16 subjects) as in Figure 1. Plot conventions are as described in Figure 1.

Memory and attentional processes assessed by the composite SAC score were improved following moderate physical exertion (1.04-fold; 27.9 ± 0.4 to 29.1 ± 0.3 mean ± SEM, Wilcoxon *p*=0.004) (Figure 2(a)). Subcomponents of the SAC were further examined to determine whether exercise-related changes were selective for a particular cognitive domain. Significant improvements in performance were limited to the delayed recall task in both genders following moderate exercise (4.3 ± 0.2 to 4.8 ± 0.1 items ± SEM, Wilcoxon *p*=0.016). Reading speed during the K-D test also improved after moderate exercise (0.9-fold reduction in total time; 43.9 ± 2.2 to 39.9 ± 1.6 (mean ± SEM total (sec), Wilcoxon *p*=0.004)) (Figure 2(b)) without an effect on card reading accuracy. However, GSC measures of effect and mental status were not different following moderate exercise (0.9-fold; 0.88 ± 0.3 to 0.81 ± 0.3 (mean ± SEM), Wilcoxon *p*=0.98) (Figure 2(c)). Vestibular function measures were also found to be unchanged after exercise (BESS: 1.1-fold increase; 7.1 ± 0.6 to 7.6 ± 0.6 mean ± SEM total errors, Wilcoxon *p*=0.30) (Figure 2(d)).

Gender was a factor in the effect of the exercise on cognitive performance and balance assessment. The effect of exercise on K-D test performance was greatest for female athletes (0.9-fold reduction from baseline; 46.9 ± 3.5 to 41.3 ± 2.6 mean ± SEM total (sec), Wilcoxon *p*=0.008). Additionally, when each gender was analyzed separately, female subjects demonstrated a significant increase in the number of errors during the BESS after moderate to hard exercise (1.4-fold increase; 5.4 ± 0.5 to 7.3 ± 0.7 (mean ± SEM errors), Wilcoxon *p*=0.008). In contrast, balance measures in male subjects were not affected by exercise. Gender did not appear as a contributing factor for measures of the SCAT3 instrument. Together, these results indicate gender may be a critical consideration when evaluating the utility of a single assessment of cognitive function.

### 3.2. Exercise Effects during EC/EO EEG Activation

**Figure 3:**
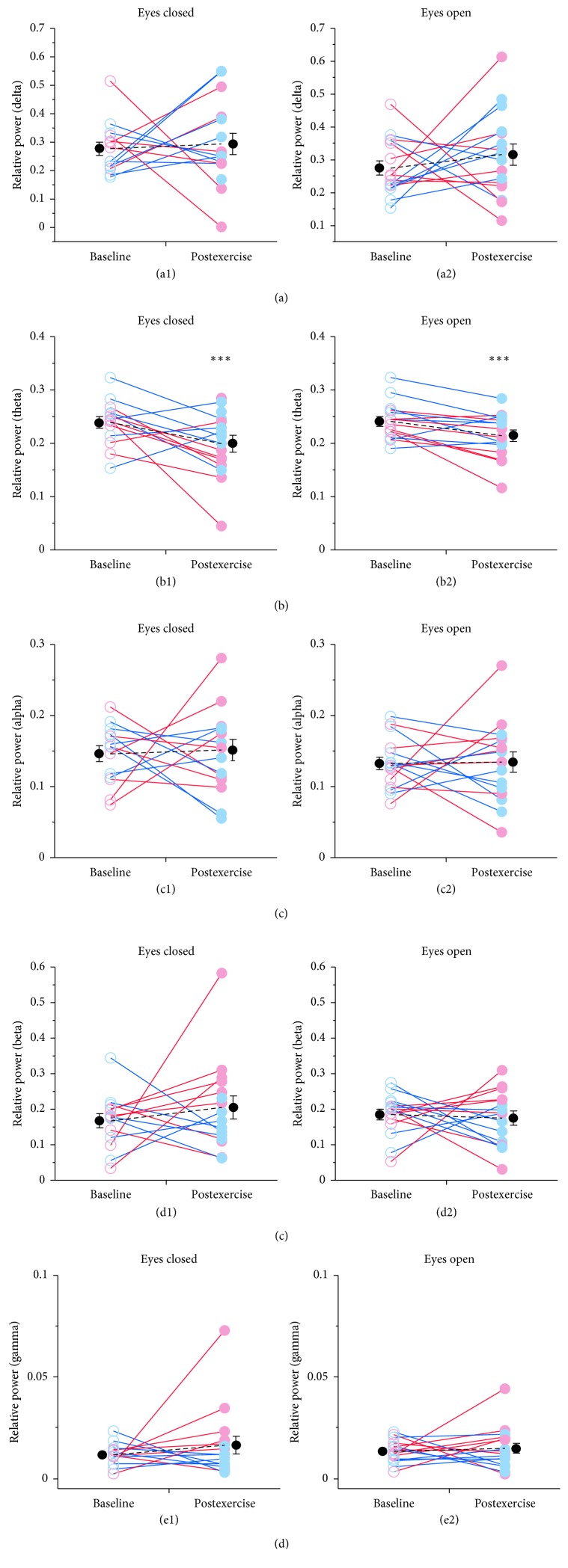
Effects of moderate exercise on qEEG relative spectral power. Spectral power was quantified for each subject during eyes closed (EC) (left; *n* = 14) and eyes open (EO) (right; *n* = 16) tasks. For each spectral band ((a) delta, (b) theta, (c) alpha, (d) beta, and (e) gamma), the mean relative power during each task is plotted during baseline (open circle) and after 1-mile run (closed circle). EEG was recorded from position Fp1 (international 10–20 montage placement system). EC data from two subjects contained too much artifact noise and were excluded from analysis. Plot conventions are as described in Figure 1.

Frontal EEG spectral power changes were first examined during the initial EC and EO components of the 14-task neural assessment. Consistent with well-established observations, a peak in spectral power was observed in the alpha frequency range (8–12 Hz) during the EC condition. Additionally, the alpha peak was significantly reduced or “blocked” during the EO task (EC 0.15 ± 0.01 versus EO 0.13 ± 0.01; Wilcoxon *p*=0.04). Acute exercise did not change relative alpha power during either the EC or EO conditions (Figure 3; Wilcoxon *p*=0.945 and *p*=0.777, respectively). However, relative theta power during both EC and EO conditions was significantly reduced after moderate exercise (EC: 0.24 ± 0.01 to 0.19 ± 0.01 mean ± SEM; Wilcoxon *p*=0.001 and EO: 0.24 ± 0.01 to 0.21 ± 0.02 mean ± SEM; Wilcoxon *p* < 0.001), and these effects of exercise on relative theta survived FDR multiple comparisons adjustment (Table 1). In contrast, moderate exercise did not affect relative or absolute measures of delta power (Figure 3; Table 1). Together, the selective actions of exercise on theta power suggest that these effects were not a result of heart rate, breathing, or sweating related artifact generated after exercise. Gender analyses indicated that theta power during EC and EO conditions tended to be higher in females than male athletes; these differences were not statistically different between genders. Interestingly, the only gender-related effects of exercise observed was a significant increase in relative beta and relative gamma power during the eyes closed task for male athletes, and these actions were dependent on %SpO_2_ levels (beta: ANOVA *F*_(7,251)_ = 2.258, *p*=0.031; gamma: ANOVA *F*_(7,251)_ = 3.117, *p*=0.004). For all remaining qEEG features examined during the EC or EO task, moderate exercise produced little effect on spectral power measures.

### 3.3. Exercise Effects on EEG Activity during Cognitive Testing

Different neural circuits are involved in supporting the cognitive functions necessary to perform each component of the SCAT3 instrument, K-D test, BESS vestibular assessment, and visual simulation tasks. These circuits can generate different oscillatory EEG frequencies that may be differentially modulated by exercise.

**Figure 4:**
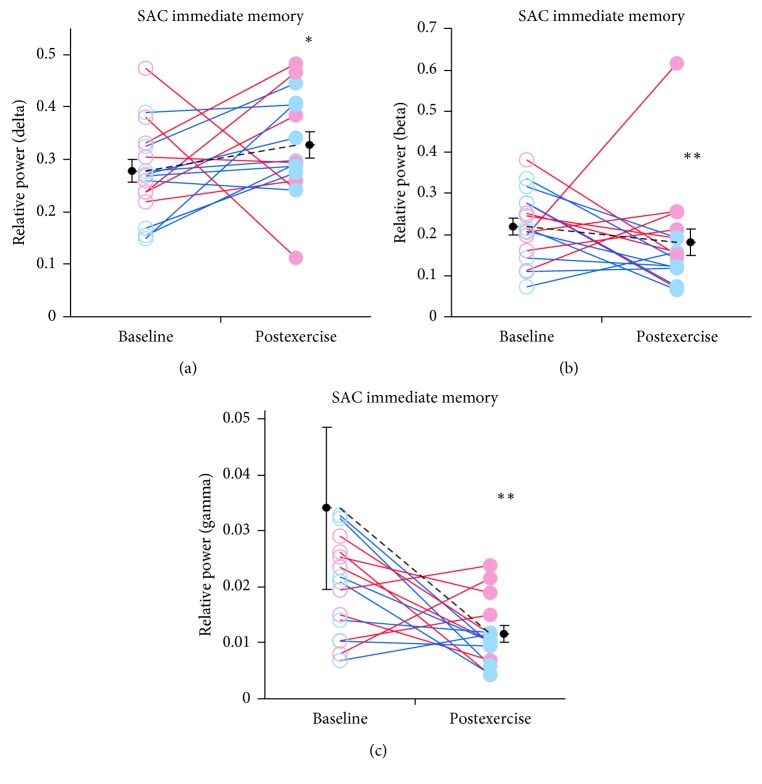
Effects of moderate exercise on qEEG relative spectral power bands during the immediate memory task of the SAC component of the SCAT3. Spectral power was quantified from each subject during each cognitive task. (a) Relative delta power was increased during the immediate memory task. (b) Relative beta power was decreased during the immediate memory task. (c) Similar to beta, gamma relative power was decreased during this task. One data point (gamma baseline value = 0.249) was not drawn for clarity. Plotting conventions are identical to Figure 1.

The majority of exercise-related qEEG changes occurred during the immediate memory task of the SAC (Table 2). During this task, relative power in the delta band was significantly increased (*n* = 16, 1.18-fold, 0.28 ± 0.02 to 0.33 ± 0.02 mean ± SEM; Wilcoxon *p*=0.017; Figure 4(a)), whereas relative power in the beta and gamma bands was decreased (*n* = 16, 0.82-fold, 0.22 ± 0.02 to 0.18 ± 0.03 mean ± SEM; Wilcoxon *p*=0.044 and *n* = 16, 0.35-fold, 0.04 ± 0.02 to 0.01 ± 0.00 mean ± SEM; Wilcoxon *p*=0.001, respectively; Figures 4(b) and 4(c)). Relative and absolute power band ratios were also significantly affected by exercise during the SAC immediate memory task. A significant increase in relative band power was observed for delta/alpha (*n* = 16, 3.29 ± 0.29 to 2.41 ± 0.29 mean ± SEM; Wilcoxon *p*=0.030), delta/beta (*n* = 16, 2.73 ± 0.32 to 1.68 ± 0.32 mean ± SEM; Wilcoxon *p*=0.033), and delta/(alpha + beta) (*n* = 16, 1.45 ± 0.15 to 0.95 ± 0.15 mean ± SEM; Wilcoxon *p*=0.037) (Supplemental Figure 2). Band ratios involving theta power were also increased including theta/(alpha + beta) (*n* = 16, 1.04 ± 0.10 to 0.74 ± 0.10 mean ± SEM; Wilcoxon *p*=0.037) and (delta + theta)/(alpha + beta) (*n* = 16, 2.49 ± 0.24 to 1.69 ± 0.24 mean ± SEM; Wilcoxon *p*=0.033). Band ratios derived from absolute spectral power measures were also increased. These included delta/beta (*n* = 16, 2.73 ± 0.32 to 1.68 ± 0.32 mean ± SEM; Wilcoxon *p*=0.033), delta/(alpha + beta) (*n* = 16, 1.54 ± 0.15 to 0.95 ± 0.15 mean ± SEM; Wilcoxon *p*=0.037), (delta + theta)/(alpha + beta) (*n* = 16, 2.49 ± 0.24 to 1.69 ± 0.24 mean ± SEM; Wilcoxon *p*=0.033) and theta/(alpha + beta) (*n* = 16, 1.04 ± 0.10 to 0.74 ± 0.10 mean ± SEM; Wilcoxon *p*=0.037) ratios. However, exercise-related decrease in relative gamma power was the only qEEG feature during this task to survive FDR multiple comparisons adjustment (Table 2).

When interactions between EEG features across a subset of tasks were examined (EC, GSC, SAC, BESS, K-D test, and ocular pursuit task), additional qEEG features were identified that were sensitive to the effects of exercise. During the K-D test, delta/(alpha + beta) band ratio power was significantly elevated after exercise (ANOVA *F*_(7,250)_ = 2.123, *p*=0.042, *t*-test *p*=0.005). During this task, theta/beta ratio power was also significantly increased from baseline measures (ANOVA *F*_(7,251)_ = 2.104, *p*=0.044, *t*-test *p*=0.002). Mounting evidence suggests that frontal theta/beta is a marker of attentional control and other executive function [8]. In contrast, a decrease in delta/(alpha + beta) band ratio power was found during the BESS_(foam)_ test after moderate exercise (*t*-test, *p*=0.043). These results indicate that, following exercise, there is a slowing of the EEG during the K-D test and a shift to higher frequencies during the balance assessment.

Gender had a significant influence on the effect of exercise on spectral power in the delta and beta bands. In female athletes, relative delta power was increased by exercise in all tasks (ANOVA *F*_(1,251)_ = 8.442, *p*=0.004; *t*-test *p*=0.001), whereas relative beta power was decreased (ANOVA *F*_(1,251)_ = 10.175, *p*=0.0016; *t*-test *p* < 0.001). Similar exercise-related effects were observed across the band ratios examined. Specifically, delta/alpha band ratio power (ANOVA *F*_(1,250)_ = 4.677, *p*=0.032; *t*-test *p*=0.027) and delta/beta ratio power (ANOVA *F*_(1,249)_ = 6.951, *p*=0.009, *t*-test *p*=0.008) were significantly increased in females following exercise. Female specific increases were also found in the combined delta/(alpha + beta) band ratio power (ANOVA *F*_(1,250)_ = 6.366, *p*=0.012, *t*-test *p*=0.004) as well as the (delta + theta)/(alpha + beta) ratio (ANOVA *F*_(1,250)_ = 6.618, *p*=0.011, *t*-test *p*=0.002) after exercise. The effect of exercise on relative band power or band ratio power in male athletes was not significant.

## 4. Discussion

Exercise has been documented to improve certain components of cognitive function [1, 4, 5]. Psychological tests and direct EEG measures of cortical function are being used with increasing frequency to assess baseline and postinjury performance of neurological function in athletes. Despite the sensitivity of these tools to identify changes in brain function, an understanding of the impact of exercise on these measures is limited. This pilot study provides a first detailed examination of the effects of exercise on performance in several standardized assessment tools used to detect concussion. These results demonstrate that, in healthy collegiate athletes, moderate to hard acute exercise improves performance measures on a number of standardized concussion assessment tools and produces an array of changes in qEEG measures of cortical activity.

During standard EEG assessment tasks (e.g., EC or EO), relative power in the theta band was significantly decreased following exercise. Frontal theta is generally associated with working memory and other “executive” processes assessed during the SCAT3 [12, 18] and K-D test [19]. Moreover, qEEG measures of cortical activity during the SCAT3 and K-D test were modulated by exercise. A significant elevation in relative delta power and reductions in beta and gamma power were found during the SAC immediate memory task. Although performance on the immediate memory task was not sensitive to the effects of exercise, qEEG measures were sensitive enough to indicate changes in cortical function. Together, these findings suggest that if these assessment tools are to be used to evaluate athletes “on-site” or immediately after play/exercise, baseline assessment should account for exercise-related changes in these measures.

### 4.1. Effects of Exercise on Cognitive and Vestibular Function

After an acute exercise challenge of a 1-mile distance run, athlete heart rates averaged 82% of HR_max_ representing a moderate to hard exercise intensity by the American College of Sports Medicine [29]. Improvements in memory, attention, and other executive cognitive processes were found, including increased cognitive performance on the SAC component of the SCAT3 and K-D test. These findings support existing studies that suggest moderate exercise improves cognitive function [2, 3, 5]. However, performance on tasks probing vestibular function (BESS) or self-reporting of symptoms assessed for concussion (GSC) was not modified by exercise. Differential sensitivities of assessment tools to acute exercise indicate that certain cognitive processes probed by these assessment tools may be more responsive to the effects of acute exercise [2].

The effects of exercise on cognitive performance may also depend on the interval between the exercise and cognitive testing [7]. In prior studies, cognitive performance measures were enhanced in similar tasks when testing occurred 10 minutes after exercise, whereas impaired performance occurred immediately following exercise [4]. Importantly, in the current study, improvements in cognitive performance were observed in tasks distributed throughout a 7–20 minute interval following completion of the exercise challenge (Figure 1). Together these findings indicate that postexercise cognitive enhancement occurs approximately 10 minutes after moderate exercise. This testing window is likely an important consideration for the assessment of cognitive function with these tools following athletic injury, given that the interval of time between injury and “on-site” concussion testing is performed within 10–20 minutes. Future studies will need to address this issue by determining an optimal testing window for the administration of assessment batteries that minimize exercise-related confounding effects.

In the current study, little evidence was found that BESS assessments of vestibular function were affected by exercise. Overall, these findings support earlier work, suggesting that exercise produces minimal effect on balance measures [30] yet contradict the larger body of findings that moderate exercise decreases balance ability [31]. The cohort of female athletes demonstrated a significant impairment of balance following exercise that was consistent with this established literature. Therefore, an explanation for the lack of effects of exercise in male athletes is likely that, for this task, this study required a larger subject pool to reveal the deleterious effects of exercise on balance.

### 4.2. Effects of Exercise on Neurophysiological Measures of Brain Function

In the current study, we found that alpha power in frontal brain regions was minimally affected by moderate exercise during either EC or EO conditions. In contrast, the broader literature suggests that exercise may increase alpha activity [7]. This discrepancy, in part, may result from differences in recording locations between the current study and others. Indeed, evidence indicates that electrode location among other factors affects the degree to which exercise impacts EEG activity in the alpha band. For example, a recent meta-analysis indicates that exercise effect sizes at frontal electrodes are more than half of those at temporal and central/parietal sites [7]. Therefore, when these factors are considered, our findings are indeed consistent with prior findings that exercise does not affect alpha power in frontal brain regions.

Theta activity following moderate exercise is generally increased in frontal regions [7, 32, 33]. Frontal theta activity has been proposed to reflect executive, higher cognitive functions including attention and working memory [34]. Additionally, elevated frontal theta has been reported during meditative and relaxed states [35]. Theta power during the earliest tasks (EC and EO) was suppressed following acute exercise. However, theta band ratios were increased after exercise during cognitive testing that included the K-D test and SAC immediate memory task. It is easy to speculate that suppression of theta activity immediately after acute exercise reflects evidence that impaired cognitive performance may occur immediately following exercise [4], whereas during cognitive tests that occurred approximately 10 minutes after exercise, theta band power was elevated and may reflect enhanced performance in these cognitive tests. Our findings that there was a significant improvement in delayed recall performance following moderate to hard exercise does provide some support for this conjecture.

One challenge with these and other exercise studies is that sweating may influence the quality of EEG recordings. Cephalic skin potentials are generated as sweat glands fill, causing electrical impedance between the dermis and epidermis to change very slowly (over many seconds). When sweat is excreted, it can decrease impedance (and increase conductance) of EEG electrodes. Decreases in electrode impedance will reduce spectral power in low frequencies (up to approximately 8 Hz) [36]. However, we found that EEG recordings during the earliest tasks (EC and EO) did not show a decrease in *both* delta and theta power (Figure 3 and Supplementary Figure 3). Therefore, we argue that although postexercise sweating may have decreased recording electrode impedance, this effect did not significantly bias the finding of this study.

In further support of the hypothesis that exercise may differentially impact cortical activation states during different tasks, we found that a number of qEEG features were affected by exercise selectively during the SAC immediate memory task. During this task, elevated delta activity and reductions in beta and gamma power suggest a slowing of cortical activity. Others have shown similar exercise-related effects [37]; however, this is the first study to directly link exercise-dependent changes in EEG activity with performance on a cognitive task.

### 4.3. Gender Impacts Outcome Measures of Standardized Tests of Cognitive Function and EEG Activity

Exercise altered several cognitive performance outcome measures frequently used to assess concussion. Overall, the K-D test was the most sensitive to the effects of exercise alone. These effects were principally driven by the increased performance in female athletes following exercise. Female athletes also made significantly more errors on the BESS test following exercise. Gender also influenced the effects of exercise on EEG measures of cortical activity. Specifically, females were the only group to show an elevation in delta power as well as a decrease in beta power following exercise. A limited number of studies have addressed whether gender can influence the effects of exercise on cognitive, affective, or behavioral tests [38]. However, our findings indicate that this may be an important factor when using standardized assessment tools immediately after exercise. Gender-related differences in these tests may lead to biases that could result in inappropriate comparisons to baseline conditions following exercise.

### 4.4. Limitations of the Current Study

In the current study, we found a significant improvement in executive function performance across several tasks following moderate to hard exercise. These included both the SAC and K-D ophthalmologic saccade tasks. Furthermore, we identified a number of qEEG features that were modulated by exercise. One limitation of this study may include the limited number of participants. However, the current findings were strong enough to survive FDR-adjusted multiple comparison tests, and similar size subject pools have been used to study the effects of exercise on EEG activity and cognitive function [39, 40]. An overall improvement in performance contrasts recent studies that suggest exercise may produce differential effects on specific cognitive tasks. Such variability may result from differences in athletic abilities of subjects. Indeed, evidence suggests that during baseline testing and during exercise, measures of cognitive performance differ between athletes and nonathletes [41] and cortical activity differs between athletes and nonathletes [42].

A single testing protocol was repeated before and after moderate exercise. This sequence of 14 tasks comprising the neural assessment protocol was carefully chosen to minimize variability of EEG recordings and allow completion of cognitive testing within a time window most likely to be used when athletes are administered these “on-site” tests. It is possible that tasks early and late in the testing protocol may have been differently affected by exercise as the heart rate and %SpO_2_ levels normalize during recovery. Accumulating evidence indicates that performance in tests administered immediately after exercise are likely impaired by exercise, whereas improved performance in cognitive tests occurs within approximately 10–20 minutes after exercise [2]. We found improvements in performance on components of the SAC and K-D test which occurred at the beginning and the end of the cognitive testing protocol (7 and 12 minutes after exercise, respectively). Performance enhancement on tasks that occurred at different time points after exercise suggests that during postexercise testing, cognitive performance and EEG measures were assessed after the acute recovery phase linked to impaired task performance. Initial recovery from the bout of acute exercise likely occurred during collection of heart rate and %SpO_2_ measures prior to cognitive testing tasks of the neural assessment. Nonetheless, future studies will need to directly correlate postexercise heart rate and %SpO_2_ levels with these cognitive and EEG-related changes and determine the optimal testing window for assessing cognitive function postexercise that minimizes any possible exercise-related recovery effects.

In the current study, improved performance in cognitive measures could have been a result of learning or practice of these tests. Generally, the largest practice-related improvements are found with perceptual speed and spatial cognitive tests, not memory and reasoning tests [43]. One of the critical components of the SCAT and K-D test is their reported test-retest reliability [19, 43, 44]. The retest reliability of the K-D test has been shown in athletes when tests are administered with similar intervals as those used in the current study (i.e., 15 minute interval [19]). Therefore, a sham exercise group was not included in the current study to verify that changes in performance in these tests and EEG measures were a direct result of exercise. Finally, the stimulus sets (word and digit lists) were randomized across sessions to minimize any learning effects. Together, this evidence supports the assumption that these tests exhibit minimal learning and practice-related changes. Furthermore, between the two testing sessions, athlete performance in only two of the tasks was improved following exercise. Such a lack of global improvement in all tasks and the relative test-retest reliability of these measures indicates that our observations are most likely a result of the effects of moderate to hard acute exercise.

EEG data were collected with a single recording dry electrode at the Fp1 position in the current study. This approach contrasts with the majority of EEG studies utilizing a full 20-lead 10/20 electrode placement system or those with even higher electrode density. These latter systems have the advantage of extracting spatial information to estimate the location of sources of EEG activity. One of the most prevalent features found in EEG recordings in the published literature is an increase in spectral power within the alpha band when quietly resting with EC versus EO. It is well known that optimal recording locations for alpha activity are in the posterior areas of the skull (typically occipital). At the Fp1 location, we observed the well-described peak in alpha band power during EC. Alpha activity from Fp1 was lower in magnitude than is typically observed in posterior regions. However, observed changes in alpha power in this study confirmed that a single channel telemetric recording approach could capture the salient and previously demonstrated characteristic EEG phenomena and reported actions of exercise on these measures. Moreover, in the current study, we were interested in examining spectral power changes related to performance of a number of different cognitive and behavioral tasks using a simpler device which could be potentially used by a broader community of physicians and field clinicians. Therefore, Fp1 was chosen to best represent cortical activity of the neuroanatomical regions actively engaged in these tasks.

## 5. Conclusions

The aim of the current study was to begin to better understand the effects of exercise on cognitive measures and EEG indices of brain activity currently used as tools used by clinicians in the assessment of concussion and other neurologic dysfunctions. Moreover, this study also investigated the impact of gender and a number of physiologic, cognitive, and vestibular variables on how exercise alters brain function. These data have revealed that gender likely plays an important role in assessing how exercise affects current standards in assessing cognitive function in cortical activity in the context of sports. Finally, we found that moderate exercise alters performance on standardized cognitive assessment tools regularly used by athletes. This evidence demonstrates the importance for future studies to account for these exercise-related effects when assessing changes in brain function resulting from putative exercise-related injuries, including concussion.

## Figures and Tables

**Table 1:** Results from statistical comparison of baseline and postexercise for qEEG spectral bands during the eyes-closed and eyes-open tasks. Wilcoxon rank sum test *Z* scores, unadjusted *p* values, and false discovery rate- (FDR-) adjusted *p* values are presented for both EC and EO tasks (columns) and for each spectral power band (rows). The only statistically meaningful difference that survived FDR occurred in the theta band (shown in **bold**).

		Eyes closed (EC)						Eyes open (EO)					
Band		Wilcoxon *Z*		*p* value		FDR-adjusted *p* value		Wilcoxon *Z*		*p* value		FDR-adjusted *p* value	
*Relative band power*													
r_delta		0.85		0.395		0.648		1.38		0.169		0.476	
r_theta		−3.29		**0.001**		**0.01**		−3.71		**<0.001**		**0.004**	
r_alpha		−0.07		0.945		0.945		−0.28		0.777		0.818	
r_beta		0.71		0.476		0.648		−0.7		0.486		0.648	
r_gamma		0.94		0.346		0.648		0.55		0.585		0.719	
*Absolute band power*													
a_delta		0.3		0.765		0.818		1.53		0.127		0.451	
a_theta		−1.72		0.085		0.424		0.89		0.376		0.648	
a_alpha		−0.71		0.477		0.648		0.89		0.376		0.648	
a_beta		1.31		0.19		0.476		0.51		0.611		0.719	
a_gamma		1.49		0.135		0.451		1.94		0.052		0.348	

**Table 2:** Statistically significant qEEG features beyond EC/EO tasks. Subject *n* and mean ± SEM spectral power are presented for each band and ratio (rows) with Wilcoxon rank sum test *S* (sum of the rank scores), *Z* scores, unadjusted *p* values (prob > |*z*|), and false discovery rate- (FDR-) adjusted *p* values. Significant differences and those surviving FDR correction are highlighted (**bold**).

		Baseline			Postexercise						
Task	qEEG feature^1^	*n*	Mean	Standard error mean	*n*	Mean	Standard error mean	*S*	*Z*	Prob > |*Z*|	FDR-adjusted *p* values
Eyes open	Theta power	16	0.241	0.004	16	0.214	0.004	165	−3.712	**0.000**	**0.011**
NATASCAT: SAC-immediate memory	Delta power	16	0.278	0.018	16	0.327	0.018	328	2.393	**0.017**	0.151
NATASCAT: SAC-immediate memory	Beta power	16	0.219	0.021	16	0.181	0.021	210	−2.016	**0.044**	0.140
NATASCAT: SAC-immediate memory	Gamma power	16	0.034	0.007	16	0.012	0.007	176	−3.298	**0.001**	**0.026**
NATASCAT: SAC-immediate memory	Delta/alpha band ratio power	16	2.413	0.294	16	3.290	0.294	322	2.167	**0.030**	0.182
NATASCAT: SAC-immediate memory	Delta/beta band ratio power	16	1.676	0.315	16	2.732	0.315	321	2.129	**0.033**	0.164
NATASCAT: SAC-immediate memory	Theta/(alpha + beta) band ratio power	16	0.735	0.102	16	1.038	0.102	320	2.092	**0.036**	0.141
NATASCAT: SAC-immediate memory	Delta/(alpha + beta) band ratio power	16	0.953	0.147	16	1.452	0.147	320	2.092	**0.036**	0.132
NATASCAT: SAC-immediate memory	(Delta + theta)/(alpha + beta) band ratio power	16	1.688	0.237	16	2.490	0.237	321	2.129	**0.033**	0.150
NATASCAT: SAC-concentration	Gamma power	16	0.020	0.002	15	0.012	0.002	167	−2.866	**0.004**	0.075
KD-test	Beta power	15	0.188	0.017	16	0.135	0.024	293	2.075	**0.038**	0.129
KD-test	Gamma power	15	0.021	0.002	16	0.014	0.003	297	2.233	**0.026**	0.173
KD-test	Theta/beta band ratio power	15	1.329	0.130	16	2.789	0.533	186	−2.115	**0.034**	0.144
Binaural 12 Hz tone	Gamma power	16	0.023	0.002	16	0.014	0.002	191	−2.732	**0.006**	0.085
Binaural 6 Hz tone	Gamma power	16	0.058	0.039	15	0.011	0.001	176	−2.510	**0.012**	0.131
Fixation task	Beta power	16	0.237	0.018	16	0.183	0.018	207	−2.129	**0.033**	0.180
Fixation task	Gamma power	16	0.022	0.002	16	0.014	0.002	203	−2.280	**0.023**	0.175

^1^All qEED features listed are derived from relative power calculations.

## Data Availability

The EEG and behavioral data used to support the findings of this study are currently under embargo while the research findings are commercialized. Requests for data, 12 months after publication of this article, will be considered by the corresponding author.

## Supplementary Materials

Supplementary Materials Supplemental Table 1: Lehigh collegiate soccer participant demographics. Supplemental Figure 1: the effects of artifact removal on EEG time series and spectral power. (A) As an example, 30-second traces of EEG data are plotted during the eyes closed (top) and eyes open (bottom) tasks after band-pass filtering between 0.5 and 45 Hz. Red lines indicate when artifact was detected by the software. These traces are replotted in the adjacent panels following artifact removal and replacement with interpolated data from preceding and training data. (B) Welch absolute power spectrums of data from (A) eyes closed. The left spectrum was generated from the raw data (with artifacts). The right spectrum was generated from denoised data. The spectra from the original and denoised data are nearly identical. Supplemental Figure 2: the effects of moderate exercise on qEEG relative spectral power band ratios during the immediate memory task of the SAC component of the SCAT3. Spectral band ratio power was quantified for each subject during each cognitive task. (A) Delta/alpha ratio power was increased during the immediate memory task. (B) Delta/beta ratio power was also increased during this task. (C) Band ratio power combinations including delta/alpha + beta power ratio was also increased during the SAC immediate memory task. (D) Similar increases in theta/alpha + beta power ratio occurred following exercise. Plotting conventions are identical to Figure 2 of the main manuscript. Supplemental Figure 3: the effects of exercise on EEG time series and power spectrum during the first test battery task (eyes closed). (A) A 30-second epoch of denoised EEG data (left) and its associated absolute power spectrum (right) collected from a single individual under baseline conditions. (B) An identical length EEG time series and associated power spectrum following acute exercise. Power in the delta range 1–4 Hz is similar between conditions, whereas theta (4–8 Hz) power is reduced postexercise. These data suggest that exercise selectively reduces Fp1 theta power during eyes closed task and that this effect is not a result of an overall suppression of low frequency power.
