# Supplementary material for: Effects of Exercise on EEG Activity and Standard Tools Used to Assess Concussion
Source: J Healthc Eng. 2019 Apr 30;2019:4794637. doi: 10.1155/2019/4794637 (PMC6515073; doi:10.1155/2019/4794637)
Supplement: Supplementary Materials — Supplemental Table 1: Lehigh collegiate soccer participant demographics. Supplemental Figure 1: the effects of artifact removal on EEG time series and spectral power. (A) As an example, 30-second traces of EEG data are plotted during the eyes closed (top) and eyes open (bottom) tasks after band-pass filtering between 0.5 and 45 Hz. Red lines indicate when artifact was detected by the software. These traces are replotted in the adjacent panels following artifact removal and replacement with interpolated data from preceding and training data. (B) Welch absolute power spectrums of data from (A) eyes closed. The left spectrum was generated from the raw data (with artifacts). The right spectrum was generated from denoised data. The spectra from the original and denoised data are nearly identical. Supplemental Figure 2: the effects of moderate exercise on qEEG relative spectral power band ratios during the immediate memory task of the SAC component of the SCAT3. Spectral band ratio power was quantified for each subject during each cognitive task. (A) Delta/alpha ratio power was increased during the immediate memory task. (B) Delta/beta ratio power was also increased during this task. (C) Band ratio power combinations including delta/alpha + beta power ratio was also increased during the SAC immediate memory task. (D) Similar increases in theta/alpha + beta power ratio occurred following exercise. Plotting conventions are identical to Figure 2 of the main manuscript. Supplemental Figure 3: the effects of exercise on EEG time series and power spectrum during the first test battery task (eyes closed). (A) A 30-second epoch of denoised EEG data (left) and its associated absolute power spectrum (right) collected from a single individual under baseline conditions. (B) An identical length EEG time series and associated power spectrum following acute exercise. Power in the delta range 1–4 Hz is similar between conditions, whereas theta (4–8 Hz) power is reduced postexercise. Thes [file 4794637.f1.zip › 4794637.f1/JHE_Figure_S3.pdf]

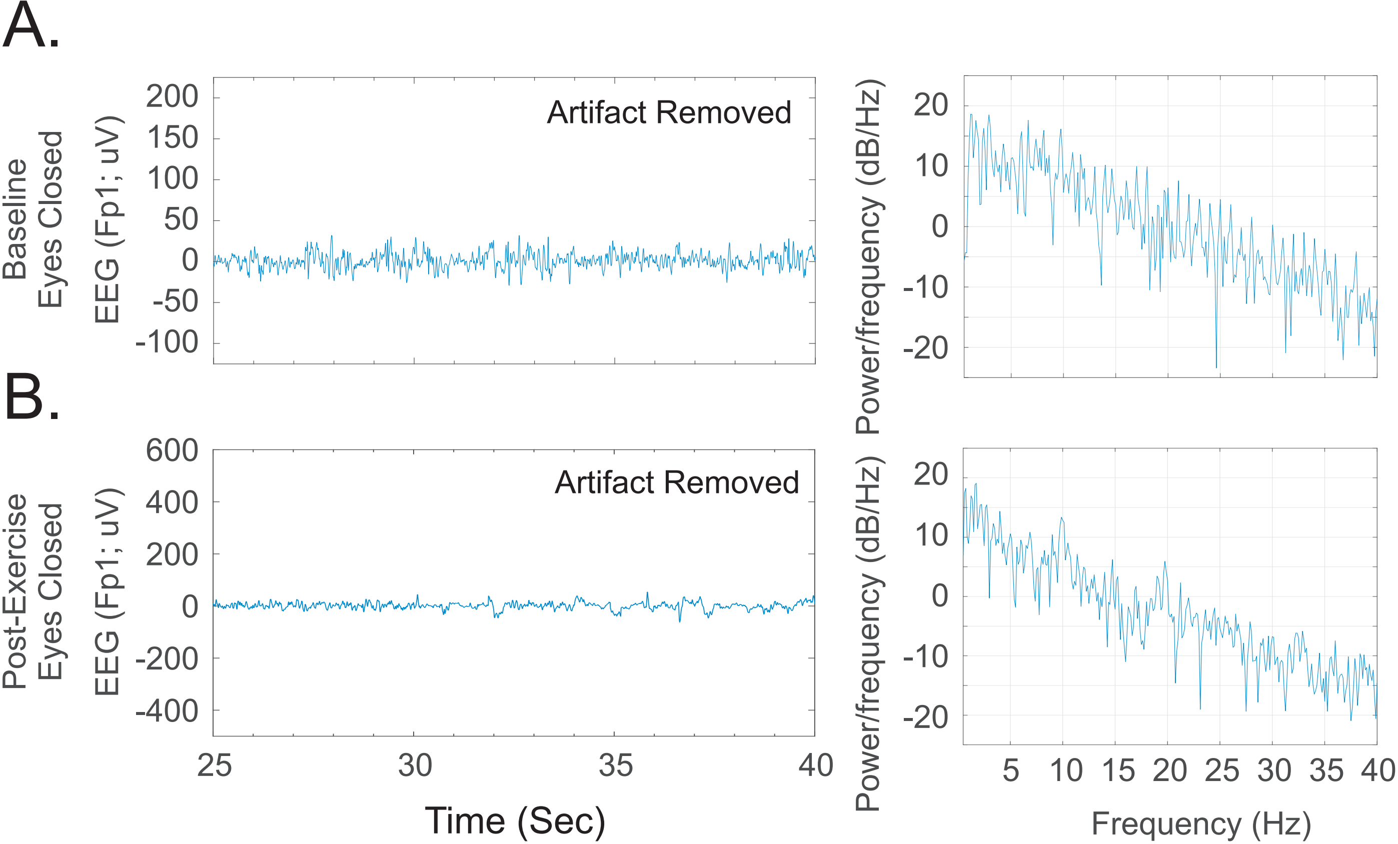

Supplemental Figure 3. The effects of exercise on EEG timeseries and power spectrum during the first test battery task (Eyes Closed). A) A 30 second epoch of denoised EEG data (left) and its associated absolute power spectrum (right) collected from a single individual under baseline conditions. B) An identical length EEG timeseries and associated power spectrum following acute exercise. Power in the delta range 1-4 Hz is similar between conditions, whereas theta (4-8 Hz) power is reduced post-exercise. These data suggest that exercise selectively reduces FP1 theta power during Eyes Closed and that this effect is not a result of an overall suppress of low frequency power.
